# A study on the prevalence of smoking habits among the student community in Aseer Region, Saudi Arabia

**DOI:** 10.3389/fpubh.2023.1257131

**Published:** 2023-12-22

**Authors:** Geetha Kandasamy, Gigi Sam, Mona Almanasef, Tahani Almeleebia, Eman Shorog, Asma M. Alshahrani, Rana A. Almohaimeed, Amjad Hmlan, Atheer Y. Al Suhaym, Kousalya Prabahar, Vinoth Prabhu Veeramani, Palanisamy Amirthalingam, Basmah Mohammed Shorog, Vasudevan Mani

**Affiliations:** ^1^Department of Clinical Pharmacy, College of Pharmacy, King Khalid University, Abha, Saudi Arabia; ^2^Department of Pharmaceutical Sciences, College of Pharmacy, Shaqra University, Dawadmi, Saudi Arabia; ^3^Department of Clinical Pharmacy, College of Pharmacy, King Saud University, Riyadh, Saudi Arabia; ^4^Eradah Hospital for Mental Health in Jazan, Ministry of Health, Jazan, Saudi Arabia; ^5^Department of Pharmacy Practice, Faculty of Pharmacy, University of Tabuk, Tabuk, Saudi Arabia; ^6^Department of Family and Community Medicine, Armed Forces Hospital Southern Region, Khamis Mushait, Saudi Arabia; ^7^Department of Pharmacology and Toxicology, College of Pharmacy, Qassim University, Buraydah, Saudi Arabia

**Keywords:** cigarette smoking, prevalence, mFTQ, awareness, quit smoking, students

## Abstract

**Objectives:**

A cross-sectional study was aimed to assess the prevalence of smoking habits among students at King Khalid University (KKU), Abha, KSA.

**Methods:**

This was a cross-sectional study using a Modified Fagerstrom Tolerance Questionnaire (mFTQ), online survey was carried out among the students of KKU. This tool uses a five-point Likert scale for all seven questions, except one question on smoking during the first 2 h of the day.

**Results:**

The prevalence of smoking among male students was 67% (*n* = 243) and females 33% (*n* = 122). Of the current cigarette smokers, 19% had a nicotine dependence score of ≥6 (high), 48% scored 4–6 (moderate) and 33% scored <4 (minimal). Association between mFTQ and the number of cigarettes per day (*p* < 0.001), first smoke of your cigarettes (*p* < 0.018), smoking in the morning (*p* < 0.007), and difficulty refraining from smoking in public areas (*p* < 0.000). The results of the current study recommend that cigarette smoking habits are a significant risk behavior among young students. The strength of this study signifies that most participants (62%) intend to quit if appropriately supported.

**Conclusion:**

According to the findings of the current investigation, smoking was quite common among males. It raises the alarm about the critical need for adequate education to support health education initiatives, discourage teen smoking, and enhance health outcomes for the community.

## 1 Introduction

Smoking is a significant global contributor to avoidable morbidity and mortality. Each year, smoking causes six million deaths worldwide. According to data from the World Health Organization (WHO), it is predicted to exceed eight million by 2030 (1). Many diseases and fatalities that may be avoided are brought on by tobacco use, in all of its forms. Around seven million individuals died worldwide in 2016 from diseases related to tobacco use, according to a 2018 report (2). By 2030, eight million people are expected to die each year from tobacco-related diseases if current trends continue (3). In spite of the fact that tobacco usage has decreased in many developed nations, 80% of the 1.1 billion current smokers who reside in these nations continue to bear the cost of tobacco-related illness and mortality (2). In addition, it was discovered that smoking rates increased statistically significantly between 1980 and 2012 in various high-income nations, including the Kingdom of Saudi Arabia (KSA) (4). From 2010 to 2014, the KSA imported tobacco goods valued at around US$ 3.4 billion (5). As a result, smoking cost the KSA 20.5 billion US dollars, and between 2001 and 2010 there were 280,000 premature deaths (6). The KSA has established specific regulations to regulate and lower tobacco consumption for the past three decades (7, 8). The use of tobacco products is prohibited at government and affiliated institutions, including college campuses, parks, shopping centers, airports, and other public areas that have been designated as tobacco-free zones. Another law taxes tobacco products at a rate of 100%. In June 2017, the most recent price rise for tobacco products went into effect (7). Nonetheless, smoking remains a serious problem among Saudi college students (9).

One of the top 10 cigarette importers worldwide is KSA. In 2010, a study of 2,564 Saudi students revealed that 8.9% of the participants were active smokers (10). Consuming tobacco is linked to a number of illnesses that can affect both sexes, including various cardiovascular, respiratory, and gastrointestinal conditions. However, reproductive system disorders specific to women, such as miscarriages, preterm birth, low birth weight, and possibly sudden infant death syndrome, are also present. Smoking has health implications on young people even though many of the harmful effects of tobacco appear later in life (11).

Tobacco is commonly used as smoking and smokeless forms of tobacco. Tobacco smoking is usually done in the form of cigarettes, pipe tobacco, and cigars. Worldwide current smokers of cigarettes are found to be 1.3 billion.

The overall prevalence of smoking is 29% of which 47.5% are men and 10.3% are women. Every year usage of tobacco is increasing by around 3.4%.

In addition reports from another study show that the prevalence rate is 13.6% among students of medicine Abha, KSA (12). Only a few studies have shown to find out the prevalence of smoking among adolescents in Saudi Arabia. The production of tobacco and its derivatives is forbidden in Saudi Arabia, By Royal Decree No. (M/56) of the Saudi Amended Anti-smoking Law, dated May 17, 2015. The term “smoking” refers to the consumption of tobacco and its derivatives, including cigarettes, cigars, tobacco leaves, tobacco molasses, and any other product containing tobacco, whether smoked as cigarettes or cigars, through the use of a pipe or shisha by chewing, sniffing or any other means. At the following locations, smoking is not permitted. (1) Mosques surrounding area and yards. (2) Ministries, government bureaus, public institutions, their branches, and other public organizations. (3) Employment spaces in businesses, organizations, factories, banks, and the government. (4) Institutions for education, health, sport, culture, and social welfare Public transportation, as defined by the implementing regulations. (5) Facilities for the production, processing, and packaging of food, beverages, and other goods for human consumption. (6) Warehouses, elevators, restrooms, and sites for the production. (7) Distribution, and refinement of petroleum, its derivatives, as well as gasoline and gas stations. (8) If these locations designate smoking zones, those areas must be separated, confined, and inaccessible to anyone who is younger than 18 years old (13).

Smoking is a significant risk factor for coronary artery disease in the Saudi population. Cigarette smoking is widespread among students of health care professionals; the study conducted in the College of Applied Medical Sciences in Riyadh, KSA reported 29% of respondents were current smokers (14). Diverse studies exhibited that smoking prevalence is relatively high among healthcare employees however they know the harmful effects of active and passive forms of smoking (7). Worldwide substantial predictors of smoking were found to be age, salary, peer pressure, family members, and academic performance (8). In addition, smoking habits in young adults were a potential predictor of smoking behavior in adulthood (9). Studies exhibited that smoking prevalence is moderately high among healthcare workers even though they know the harmful effects of active and passive smoking. A significant amount of work has been published on the predictors of smoking, but not specifically within the Saudi Arabia population. In specific, smoking by adolescents was a strong predictor of smoking behavior in young adults. Hence the present study is aimed to assess the prevalence of smoking habits among the students at KKU, Abha, KSA.

## 2 Materials and methods

### 2.1 Study site and sample size

A prospective cross-sectional study was conducted among KKU students. One thousand four hundred students approached and 731 responded with a completed questionnaire. Among those 731 questionnaires, eight due to insufficient data were excluded. Out of 723 respondents 365 were smokers and 358 were non-smokers. Three hundred sixty-five smokers was included in the present study. The participants were gathered using a snowball sampling technique.

### 2.2 Population criteria

Students of KKU who were willing to participate in the study voluntarily were included. Informed consent was obtained from the student participants who were willing to participate in the study, after explaining the project objectives. The study excluded those who did not provide their consent and had incomplete questionnaires.

### 2.3 Questionnaire

A well-structured, pre-validated nine self-administered online questionnaire (Google docs) was used. Students have responded to the modified Fagerstrom Tolerance questionnaire (mFTQ) in English. The responses were downloaded in an Excel sheet, which asks about their sociodemographic details, and social habits, especially tobacco consumption. The m FTQ is a widely used instrument to assess nicotine dependency (15). It consisted of seven questions related to the use of cigarettes, with scores ranging from 0 to 9. Question 1. “How many cigarettes a day do you smoke?” “2. Do you inhale?” “3. How soon after you wake up do you smoke your first cigarette?” “4. Which cigarette would you hate to give up?” “5. Do you find it difficult to refrain from smoking in places where it is forbidden (church, library, movies, etc.)?” “6. Do you smoke if you are so ill that you are in bed most of the day?” “7. Do you smoke more during the first 2 h than during the rest of the day?” All seven of the questions on this questionnaire use a five-point Likert scale, with the exception of the one about smoking during the first 2 h of the day. A score of mFTQ 0–2 suggests no nicotine dependence, a score of 3–5 suggests moderate dependence, and a score ≥6 usually indicates high dependence (15). Additional question about willingness to quit smoking among current smokers was added. The validation of mFTQ with salivary cotinine among adolescents found that the mFTQ scale (*r* = 0.40) is valid and applicable to smokers of adolescents (16). The participants were asked if they had any thoughts of giving up smoking, and their yes/no responses were recorded. This study, REC No. 2018-06-42, HA-06-B-001 was exempted by the KKU ethics committee.

### 2.4 Statistical analysis

One thousand four hundred people were approached, and 731 returned the questionnaire with completed responses. Unfortunately, eight questions were disqualified because the data was not full. The sample characteristics were assessed using descriptive statistics. The significant relationship between the mFTQ variables was evaluated using the chi-square test. Statistical significance was set at *p* < 0.01.

## 3 Results

Among 365 smokers, 243 (67%) were male and 122 (33%) were female. The overall prevalence of current smokers in the study was 50.48% and 49.51% were non-smokers. The total prevalence of current daily smokers in the 18–20 age group was 30% (*n* = 110), followed by 25% (*n* = 91), 25% (*n* = 91), 23% (*n* = 84), 10 (*n* = 37), and 12 (*n* = 43) in the 21–22, 25–30, and above 30 age groups. Details are given in Table 1.

**Table 1:** Age and gender wise distribution among the study population (*n* = 365).

**S. No**	**Age**	**Percentage (*n*)**
1	18–20	30 (110)
2	21–22	25 (91)
3	23–24	23 (84)
4	25–30	10 (37)
5	Above 30	12 (43)
**Gender**		
7	Male	67 (243)
8	Female	33 (122)

### 3.1 Nicotine dependence

The scoring system of the mFTQ was used in the study. The percentage of respondents who indicated they were dependent on nicotine in response to particular questions is as follows. Several cigarette smoking in a day ranges: among the current smokers 56 (15%) were found to smoke over 26 cigarettes a day, 84 (23%) were found to smoke about 16–25 cigarettes a day, 225 (62%) were found to smoke < 15 cigarettes a day. About 267 (73%) were found to inhale cigarette smoke always and 98 (27%) of smokers never inhaled cigarette smoke. A total of 157 (33%) participants said they smoke within the first 30 min of waking up, and 57% (208) said they smoke after awakening but before midday, and more than 30 min.

A total of 162 (44%) were found to dread giving up their first smoke of the day and 203 (56%) were found to give up any other cigarette in the afternoon. One hundred and eighty-one (180) respondents (49%) found it difficult to abstain from smoking when they were ill and spent the majority of the day in bed, and 61% (221) of smokers stated they find it difficult to stop from smoking in public areas where it is prohibited (Mosque, libraries, etc.). Also, 49% (180) of respondents were discovered to smoke more during the first 2 h of the day. Out of 365 smokers, 216 (59%) smokers were willing to quit and 149 (41%) smokers are not willing to quit. The details are given in Table 2. mFTQ (*n* = 365) scores of 0–2 indicate no dependence, 3–5 indicate moderate dependence, and ≥6 indicate substantial dependence. Mean and standard deviation of no dependence 1.35 ± 0.74, moderate dependence 3.91 ± 0.81, substantial dependence 6.62 ± 0.72. The number of current smokers in each category; no dependence, moderate dependence, and substance dependence were 121 (33%), 175 (48%), and 69 (19%) respectively. The details are given in Table 3.

**Table 2:** Response to Modified Fagerstrom Tolerance Questionnaire (*n* = 365).

**S. No**	**Modified Fagerstrom Questions (mFTQ)**	**Responses**	**Number (%)**
1	Number of cigarettes smoking in a day	Over 26 cigarettes a day	56 (15)
		About 16–25 cigarettes a day	84 (23)
		Less than 15 cigarettes a day	225 (62)
2	Inhalation of cigarette smoke	Always	267 (73)
		Never	98 (27)
3	First smoke on waking up	Within the first 30 min	157 (43)
		More than 30 min after waking but before noon	208 (57)
4	Cigarette would you hate to give up	First cigarette in the morning	162 (44)
		Any other cigarette in the afternoon	203 (56)
5	Difficult to refrain from smoking in public	Yes	221(61)
		No	144 (39)
6	Difficult to refrain from smoking when sick	Yes	180 (49)
		No	185 (51)
7	More smoking in first 2 h of the day	Yes	180 (49)
		No	185 (51)
8	Willingness to quit	Yes	216 (59)
		No	149 (41)

**Table 3:** Scores of Modified Version of the Fagerstrom Tolerance Questionnaire (mFTQ) (*n* = 365).

**S. No**	**Scores**	**Mean ±SD**	**Number of respondents (%)**
1	0–2 no dependence	1.35 ± 0.74	121 (33)
2	3–5 moderate dependence	3.91 ± 0.81	175 (48)
3	≥6 substantial dependence	6.62 ± 0.72	69 (19)

SD, standard deviation.

### 3.2 Nicotine dependence and variables of Modified Fagerstrom Tolerance Questionnaire (Chi Square Test)

Relationship between mFTQ and the number of cigarettes per day among the respondents (*p* < 0.001). Relationship between mFTQ score and first smoke of your cigarettes (*p* < 0.018). Relationship between mFTQ and smoke in the morning among the respondents (*p* < 0.007). Relationship between mFTQ and the difficulty refraining from smoking in public areas among the respondents (*p* < 0.000). Correlation between mFTQ and the cigarettes would you hate to give up among the respondents (*p* < 0.202). The details are given in Table 4.

**Table 4:** Nicotine dependence and variables of Modified Fagerstrom Tolerance Questionnaire (mFTQ).

**S. No**	**Variables**	***p*-value**
1	mFTQ score and number of cigarettes per day	0.001^*^
2	mFTQ score and first smoke of your cigarettes	0.018^*^
3	Do you smoke in the morning	0.007^*^
4	Difficult to refrain smoking from public area	0.000^*^
5	Cigarette would you hate give up	0.202

^*^p < 0.01 is statistically significant.

Chi Square Test shows the association between the variables of questionnaire and Nicotine dependence among the study population.

## 4 Discussion

Out of 723 total respondents, 365 respondents were found to be smokers and 358 were non-smokers. Among 365, 243 (67%) were male and 122 (33%) were female. The overall prevalence of current smokers in the study was 50.48%. Cigarette smoking is more predominant in Saudi males, the majority started smoking during their adolescent period and continued thereafter for many years (17). In addition, according to Wali's 2011 research, 9.1% of women and 24.8% of men smoke cigarettes (13).

Another study conducted in Saudi Arabia reported prevalence among male students was 30.4% (18). Moreover, a study on smoking rates among students at King Faisal University in Saudi Arabia found a similar prevalence of 28.1% (19). The prevalence of smoking among male students was high in the present study and female smokers are less compared to males. In the present study, among female students, 33% had recently used cigarettes, It is comparable to the research done to establish how the tobacco epidemic was spreading among female university students in Jeddah, Saudi Arabia's Western Region (20).

### 4.1 Measurement of nicotine dependence

The study made use of the mFTQ scoring system. The proportion of respondents who responded favorably to particular questions about nicotine dependence. According to the study's findings, 62% of respondents smoked fewer than 15 cigarettes per day, 33% smoked between 16 and 25 cigarettes, and 15% smoked more than 26 cigarettes per day. This indicates the responders' high level of nicotine dependence. Nicotine dependency is directly correlated with this high daily cigarette usage (21).

About 73% of respondents were found to inhale cigarette smoke always and 27% of smokers never inhaled cigarette smoke. In total, 43% of subjects admitted to smoking within the first 30 min of waking up, and 57% said they smoke before midday, more than 30 min after awakening. This could be attributed to the fact that the plasma nicotine levels are short and half-lived so it declines immediately after they wake up. This may be the reason that they are tempted to smoke early in the morning (22). Consequently, a morning cigarette urge is one of the signs of nicotine dependence as reported by Lamin et al. (23).

A total of 162 (44%) were found to many find it difficult to put up their morning cigarette, and 203 (56%) were found to give up any other cigarette in the afternoon. In the same way, 54.0% of smokers answered hate to give up the smoke during the first 2 h of the day (18). Sixty-one percentage (221) of smokers said that they find it difficult to refrain from smoking in public places where it is forbidden (Mosque, libraries, etc), 49% (180) of the respondents found it difficult to refrain from smoking when they are ill, they spend the majority of the day in bed, and 49% (180) of the respondents were found to more smoking in first 2 h of the day (24). Seventy percent of smokers reported that it is difficult not to smoke in forbidden areas like mosques and libraries. When severely ill (and spending the majority of the day in bed), those who were diagnosed as full smokers said they would smoke (25).

mFTQ (*n* = 365) scores of 0–2 indicate no dependence, 3–5 indicate moderate dependence, and ≥6 indicate substantial dependence. Scores of no dependence, moderate dependence, and substance dependence were 121 (33%), 175 (48%), and 69 (19%), respectively. Similar results were reported by other studies that moderate nicotine dependence is high (26). Similar findings from earlier studies indicated that Thiruvananthapuram, Kerala's rural population had a high level of mild nicotine dependence (27). Another cross-sectional study revealed that among smokers, 34.4%, 39%, and 26.5% have no or mild, moderate, or severe nicotine dependency, respectively (28). According to a study conducted in Nepal, smokers with low degrees of nicotine dependence (4) had nicotine levels of 70.2%, medium (4–6), 52.8%, and high (7–10), 12.0% (29).

### 4.2 Nicotine dependence and variables of Modified Fagerstrom Tolerance Questionnaire (Chi Square Test)

From the study outcomes, there was a strong correlation found between mFTQ scores and the number of cigarettes per day, mFTQ scores and first smoke of cigarettes, mFTQ scores and smoking habit in the morning, mFTQ scores and difficulty to refrain smoke from public areas. The findings of the present study explain that the higher number of cigarettes per day has a significant association with the mFTQ scores (23). Previous study shown that the mFTQ score often increased as the severity of the smoking is increased due to a strong linear connection between the two variables (*p* < 0.001) (25).

### 4.3 Quitting behavior

Out of 365, 216 (59%) smokers were willing to quit and 149 (41%) smokers are not willing to quit. This shows that college students in the KSA may be aware of the advantages of quitting smoking, and that in the future, effective education initiatives will be required to support them. This result is congruent with those of Majmaah University KSA where 71.8 percent of smokers in college had previously tried to give up smoking (18). Another study conducted in KSA revealed that 65% of smokers were ready to give up the habit (30). According to other studies, the majority of smokers had made at least one attempt to stop smoking (31). The readiness to give up smoking among patients in all categories, including those with mild, moderate, and severe nicotine dependence (28). After receiving smoking cessation education in schools, knowledge and attitudes about smoking harm prevention considerably improved. Numerous research have shown this improvement in awareness and attitudes toward smoking harm prevention. The organization plan activities that can improve the physical fitness and cardiopulmonary endurance of outpatients who are quitting smoking in clinics, as well as a series of smoking cessation education and training sessions (32).

The high prevalence of tobacco use and the affirmative responses provided by respondents who were smokers to the Fagerstrom questions suggest that tobacco use is becoming a serious issue in universities. The ambitious tobacco control initiatives developed by the health ministry must be implemented locally. The strict enforcement of the laws adopted with that purpose and a reduction in the supply and demand for tobacco products should be the objectives of the policies (33, 34) and antismoking programs.

### 4.4 Limitations

This type of cross-sectional study has limitations, including recall bias. Moreover, the study's generalizability may be limited because its participants were college students from a single public university.

## 5 Conclusion

Almost 50% of smokers in this study had a moderate level of dependency. Overall, 59% of smokers expressed a willingness or intention to stop smoking. The present study concludes that the prevalence of smoking habits in males was relatively high. Women were less likely than men to smoke every day. In this study, around 50 % of smokers were moderately dependence. It raises the alarm about the critical need for adequate education to support health education initiatives, discourage teen smoking, and enhance health outcomes for the community. To make sure that tobacco control measures are effective and efficient, university anti-smoking policies should be evaluated on a regular basis. In addition, we require programs for quitting smoking, health promotion, and education about the adverse effects of smoking.

## Data availability statement

The raw data supporting the conclusions of this article will be made available by the authors, without undue reservation.

## Ethics statement

This study, REC No. 2018-06-42, HA-06-B-001 was exempted by the KKU Ethics Committee. The studies were conducted in accordance with the local legislation and institutional requirements. The participants provided their written informed consent to participate in this study.

## Author contributions

GK: Conceptualization, Data curation, Funding acquisition, Methodology, Writing – original draft, Writing – review & editing. GS: Writing – review & editing, Data curation, Methodology, Supervision. MA: Writing – review & editing, Conceptualization, Formal analysis, Investigation, Software. TA: Formal analysis, Project administration, Validation, Writing – review & editing. ES: Resources, Software, Validation, Visualization, Writing – review & editing. AMA: Project administration, Writing – review & editing. RA: Project administration, Validation, Writing – review & editing. AH: Formal analysis, Writing – review & editing. AYA: Data curation, Supervision, Writing – review & editing. KP: Supervision, Writing – review & editing. VV: Methodology, Writing – review & editing. PA: Formal analysis, Validation, Writing – review & editing. BM: Conceptualization, Data curation, Methodology, Writing – review & editing. VM: Conceptualization, Data curation, Project administration, Software, Supervision, Validation, Writing – original draft.
